# Fast CNN Stereo Depth Estimation through Embedded GPU Devices

**DOI:** 10.3390/s20113249

**Published:** 2020-06-07

**Authors:** Cristhian A. Aguilera, Cristhian Aguilera, Cristóbal A. Navarro, Angel D. Sappa

**Affiliations:** 1 Universidad Tecnológica de Chile INACAP, Av. Vitacura 10.151, Vitacura 7650033, Santiago, Chile; 2 Departamento de Ingeniería Eléctrica y Electrócnica, University of Bío-Bío, Concepción 4051381, Chile; cristhia@ubiobio.cl; 3 Institute of Informatics, Universidad Austral de Chile, Valdivia 5111187, Chile; cnavarro@inf.uach.cl; 4 Escuela Superior Politécnica del Litoral, ESPOL, Campus Gustavo Galindo, Guayaquil EC090101, Ecuador; asappa@ieee.org; 5 Computer Vision Center, Edifici O, Campus UAB, Bellaterra, 08193 Barcelona, Spain

**Keywords:** stereo matching, deep learning, embedded GPU

## Abstract

Current CNN-based stereo depth estimation models can barely run under real-time constraints on embedded graphic processing unit (GPU) devices. Moreover, state-of-the-art evaluations usually do not consider model optimization techniques, being that it is unknown what is the current potential on embedded GPU devices. In this work, we evaluate two state-of-the-art models on three different embedded GPU devices, with and without optimization methods, presenting performance results that illustrate the actual capabilities of embedded GPU devices for stereo depth estimation. More importantly, based on our evaluation, we propose the use of a U-Net like architecture for postprocessing the cost-volume, instead of a typical sequence of 3D convolutions, drastically augmenting the runtime speed of current models. In our experiments, we achieve real-time inference speed, in the range of 5–32 ms, for 1216 × 368 input stereo images on the Jetson TX2, Jetson Xavier, and Jetson Nano embedded devices.

## 1. Introduction

Depth estimation from stereo cameras is an essential cue for many robotic applications, (e.g., object manipulation [1], obstacle avoidance [2], and 3D object detection [3]). It gives, for example, a robot the ability to perceive and interact with 3D objects and environments, which is critical for real-world applications. In essence, depth estimation from a stereo vision system is a correspondence problem, where for each pixel in a reference image, we need to find its corresponding one in a target image. The relative distance between two corresponding pixels is called disparity, and it is directly related to depth.

Stereo disparity estimation is a classic problem that, for decades, has attracted interest from numerous researchers around the world. From initially handcrafted solutions to the newer convolutional neural network (CNN) approaches, stereo disparity estimation solutions have now reached incredible subpixel accuracy in multiple available benchmarks (e.g., [4]). Subpixel accuracy comes at the cost of compute-intensive operations that, to achieve real-time performance, require the use of high-end graphic processing units (GPUs) which, although considered energy-efficient, are not always suitable for all types of robots and applications. In the current work, the term real-time will refer to disparity maps obtained in less than 33.3 ms—i.e., at least 30 frames per second (FPS) as in [5]. In other words, this implies processing at the speed of the cameras, which is useful for many robots and computer vision applications.

Accomplishing disparity subpixel accuracy on small robots and edge applications is currently a hard task. Although the newer embedded GPU devices have helped to close the gap to desktop GPU performance, the difference in TFLOPs is still a disadvantage for embedded GPUs. Therefore, highly accurate models designed for high-end desktop GPUs today cannot run under real-time constraints on current embedded GPU devices. Moreover, the performance of newer lightweight models on different embedded GPU devices has only been partially explored. Hence, one of the goals of this work is to assess the performance of current CNN-based stereo disparity models on embedded GPU devices, using conventional optimization techniques and custom CUDA kernels. Additionally, we propose a crucial design change to current state-of-the-art models to achieve faster runtime speed on current embedded GPU devices.

The main contributions of this work are as follows:

- We assess the performance of two of the fastest state-of-the-art CNN-based models to compute stereo disparity on embedded GPUs, in order to know their limitations for further real-time applications (e.g., small robotics platforms). In this study, to have a common development platform, three of the most novel NVIDIA embedded GPUs have been selected (from the cheapest Jetson Nano to the more expensive Jetson Xavier).
- We evaluate the state-of-the-art models using a custom CUDA kernel for cost-volume computation and the TensorRT SDK for network layer inference optimization showing a more realistic runtime performance on current embedded GPU devices. State-of-the-art evaluations usually do not consider model optimization techniques under their evaluations, leaving unknown what is the current potential on embedded GPU devices.
- We propose the use of a U-Net [6] like architecture for cost-volume postprocessing, instead of the typical sequence of 3D convolutions. Using an U-Net like architecture for the postprocessing step has two key benefits: (1) The network can run much faster since 3D convolutions are costly operations on embedded devices, and (2) Having a faster inference model allows for the computation of disparity models at a higher resolution affecting the disparity pixel error performance of the network directly.

The remainder of this article is organized as follows—Section 2 reviews previous works related to CNN-based stereo disparity estimation models. Section 3 describes the embedded devices used in this work. Section 4 evaluates two fast CNN-based models on three different embedded GPU devices, showing the current status of disparity estimation on those devices. Section 5 evaluates the same networks from Section 4 but applies optimization techniques over the models having a more accurate view of the performance on current embedded GPU devices. Based on the results from Section 5, in Section 6, we propose a net model to achieve state-of-the-art runtime and disparity pixel error performance (Source code and trained models are available at www.github.com/ngunsu/festereo). Section 7 discusses the result of our proposed model. Finally, in Section 8, the conclusion of our work is presented.

## 2. Related Works

The estimation of depth using stereo images is a well-known problem that has attracted the attention of many researchers for several decades. In the last few years, the accuracy of the estimations based on convolutional neural networks has outperformed traditional approaches such as [7] thanks to the use of novel and demanding computational solutions that run on GPUs (e.g., [4,8,9,10]).

Learning-based depth estimation solutions are closely related to classical solutions, where learned modules replace carefully engineered modules, e.g., cost volume computation. In early successful results, Zbontar and Lecun [8] trained a network to learn a similarity measure between small input patches to estimate the stereo matching cost between pixels of both images: the first stage in many traditional algorithms [11]. Similarly, Luo et al. [9] used a network to compute the stereo matching cost in the form of a classification problem, where the output category is the disparity of the corresponding pixel. Although the Luo et al. [9] result was not as accurate as the one from Zbontar and Lecun [8], it was much faster because the solution required convolving the trained weights just once per image. Other approaches attempt to tackle the postprocessing stage, like Jie et al. [12], where the authors train a recurrent model to perform left-right consistency, which is an effective postprocessing method to enhance the disparity estimation. Similarly, Batsos and Mordohai [13] propose a network model to refine disparity maps using a recurrent and residual CNN architecture.

One goal in many CNN-based solutions nowadays is to remove handcrafted algorithms from the processing pipeline and try to solve the problem from the beginning to the end with a single model (end-to-end models). In this context, Kendall et al. [14] propose a model that outputs the disparity of two rectified input images. In essence, the model uses a siamese network to compute the features of each pixel and 3D convolutions and deconvolutions to compute the final cost volume. The last step consists of a soft argmin operator that allows the network to have subpixel precision. Chang and Chen [10] follow a similar approach, introducing an additional spatial pyramid pooling module at the feature computation step, that provides more object context information to the network. Previously described methods aim for accuracy rather than runtime speed, and their application to embedded GPU devices in real-time environments is not possible, due to the computational and memory cost.

CNN-based stereo depth estimation in real-time using CNN is a hard task due to the necessity of reducing the number of model parameters and computations. Currently, the most adopted solution has been to downsample the input image through max-pooling operations, computing stereo disparity at a lower resolution, and later upsampling the disparity map. Khamis et al. [15] estimate the depth at a lower resolution and later refine it at a larger scale. The model has two parts: (1) depth estimation and (2) refinement. The first part of the network is a siamese network that helps to compute depth at a lower resolution, which is fast. The second part refines and upsamples the initial estimation, avoiding one of the most significant bottlenecks in in-depth estimation: the computation of the cost volume, which depends on the number of possible disparities that at lower resolution are less. In this line, Wang et al. [5] propose an iterative way for estimating depth from stereo-images to assure real-time performance. The idea is to estimate the depth at a small resolution and then refine the solution until there is no more time available, keeping real-time performance at an unknown accuracy. We evaluate both models in the next section.

Regarding fast classical solutions that can run on embedded GPU devices, it is necessary to mention the work of Hernandez et al. [16] that presents an optimized implementation of the classic Semi-Global Matching (SGM) algorithm [7] that runs in real-time on a Tegra TX1 GPU. The solution can run in real-time using multiple GPU devices having a similar performance, in terms of pixels error, with current fastest learning-based models.

## 3. Embedded GPU Devices

In the last few years, embedded GPUs have become quite popular. Embedded GPUs are particularly useful for building applications for autonomous machines, autonomous driving, and in general, for applications on the edge, i.e., doing computation on the device itself instead of relying on a more powerful computer connected through an external connection.

Embedded GPUs and desktop GPUs available are quite similar, but with different design goals. Embedded GPUs need to be small and power-efficient, in contrast to current desktop GPUs that usually are bigger and more power demanding. Since energy is a factor, embedded GPU devices mostly use ARM processors instead of x86 or x64 processors in contrast to conventional desktops and laptops. Additionally, embedded GPU devices, at least the current ones from NVIDIA, share memory between the CPU and the GPU, in contrast to desktop GPUs which have their own memory space with their own memory technology (HBM2, GDDR6). Table 1 shows a brief description of the embedded devices used in this work and their crucial hardware specifications.

Embedded GPUs are not powerful enough to train deep learning models but are ideal for model inference. The inference speed in each embedded device depends on its software support, which is directly related to its hardware specification and, in the case of NVIDIA devices, its CUDA supported architecture.

## 4. Disparity Estimation on Embedded GPUs

In the current work, we evaluate the two fastest models that can run in real-time, according to the literature, on one or more embedded GPU devices. In particular, we evaluate anytime-stereo from [5] and stereonet from [15], which are flexible models that can trade off between disparity pixel error estimation and runtime speed. The goal is to assess how well current solution models perform on the different available platforms, measuring the disparity error over three pixels [17] and the runtime speed in milliseconds (ms). The error over three pixels (err>3) is computed as follows:

(1) $$diff\left(x\right)=\left\{\begin{array}{cc} 1, & \mathrm{if}\;|x|>3\\ 0, & \mathrm{otherwise} \end{array}\right.$$

(2) $$err>3=\frac{\sum_{x,y}diff\left(L_{d}\left(x,y\right)-GT_{d}\left(x,y\right)\right)}{\#GT_{d}}$$

where $L_{d}$ is the estimated disparity, $GT_{d}$ is the groundtruth disparity, and *x* and *y* the pixels’ coordinates in the image.

As described in Section 2, in essence, anytime-stereo and stereonet models are similar, but they are significantly different in the way that each model applies the multiple steps of disparity estimation improvement. Anytime-stereo computes disparity maps at different resolutions, starting at 1/16 of the original resolution, and ending at 1/4 of the original resolution. As a result from this piramidal approach, a disparity estimation value on anytime-stereo depends on the value estimated at the previous resolution, the first one being the least accurate, and the last one being the most accurate. Stereonet computes a disparity map at a given resolution, in this case, 1/16 of the original one, and later applies multiple refinement steps to the disparity estimation. The number of refinement steps in stereonet is directly related to the performance of the network and its speed.

Anytime-stereo and stereonet models were implemented in Pytorch 1.3 following the same training setup parameters described in their research articles, using the same amount of data, to have a fair evaluation. Both models were firstly trained on SceneFlow [18] for ten epochs and later fine-tuned using the Kitti stereo dataset of 2012 [17] for at least 200 epochs, as in [5]. We use 10% of the training data for validations, ending the training after 30 epochs without improvement on the validation set (early stopping [19]). Runtimes are the average of at least 100 runs over stereo images of size 1216 × 368, always having a standard deviation of at least 100 times smaller than the mean. No operating system optimization was used in any of the embedded devices, running the default operating system and software. Each card uses the same SDK, jetpack 4.3, and runs the same Pytorch 1.3 version. We follow this setup in all the experimental evaluations.

Table 2 shows the disparity error and runtime performance of the different models in the three different embedded GPU devices evaluated in the current work.

**Figure 1:**
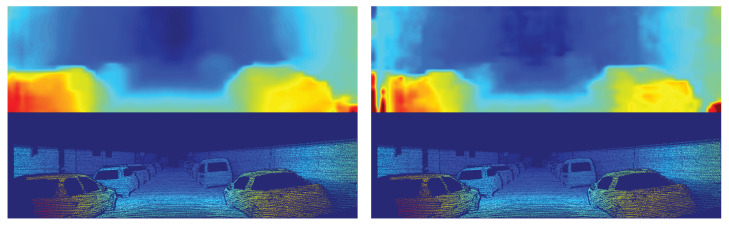
Disparity inference over the image pair 172 of the Kitti 2012 dataset [17]. (**Left**) disparity estimation using anytime-stereo stage 1. (**Right**) disparity estimation using stereonet. The disparity groundtruth is at the bottom of each image.

From the devices, the Jetson Xavier was the only one able to run all models at real-time speed, in contrast with the two other embedded devices that are less powerful and cheaper. The Jetson Nano could not run any model in real-time, and the Jetson TX2 is close to real-time performance using just the first stage of anytime-stereo, but at this stage the error can be considered too high for current standards. It is essential to mention that although anytime-stereo gives a high error on stage 1, the resulting stereo map is smooth, lacking mostly on small details as a consequence of computing the disparity map at low resolution (see Figure 1 (left)). In the case of stereonet, no refinement steps were applied to be able to run in real-time, resulting in a low accurate disparity estimation (see Figure 1 (right)).

The runtime performance observed for these models, like the one presented in their original articles, is not representative of the true capabilities of these embedded GPU devices. Embedded devices require custom kernels and optimization techniques to achieve the full potential of the stereo disparity models. In the next section, we re-evaluate these models using a custom kernel to compute the initial cost-volume and using the TensorRT SDK to optimize the performance of the network layers.

## 5. Model Optimization on Embedded GPUs

It is essential to optimize models in order to obtain their highest performance once they are used on embedded devices. Usually, embedded devices do not have resources to spare, and the implementation of custom GPU kernels and inference optimization techniques become crucial. In this context, we implement two optimizations to the models evaluated in the previous section to see the actual capabilities of the models on current embedded GPU devices. Each optimization is listed below.

1. **Custom CUDA kernel for initial cost volume computation:** cost-volume computation is essential to relate information between the left and right images in current stereo disparity models. Essentially, it consists of comparing pixels features between one position in a reference image and the features of all possible positions in the target image (maximum disparity) using a given metric: in this case, L1 distance. The cost-volume computation involves multiple loop operations that run slow using current deep learning frameworks but can be easily improved using custom GPU kernels. We follow a similar implementation to [20]. Algorithm 1 contains the pseudocode of our implementation.
**Algorithm 1:** Pseudocode of cost-volume computation GPU kernel
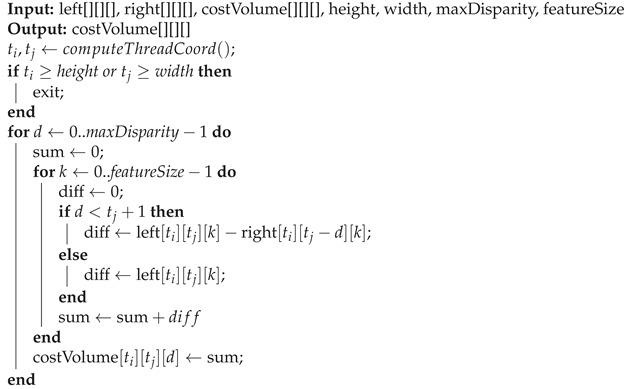

2. **TensorRT layer optimization:** TensorRT is an NVIDIA SDK library to optimize the inference time of trained models. It has several attributes, such as layer fusion, which optimize GPU memory and bandwidth by fusing common layers combination into a single kernel, and weight and precision calibration, which can quantize network weights to a lower precision like INT8. In this work, we use TensorRT 6.0.1 without reducing the precision of the model. We choose to maintain FP32 weights for two reasons. Firstly, current TensorRT SDK fully supports FP32 and FP16 operations, partially supporting other precision modes (see Table 3, top). Secondly, lower precision modes are not supported or optimized for all embedded devices, depending on their CUDA architecture (see Table 3, bottom). As a side note, we tried FP16 also without noticing any substantial improvement in the Jetson TX2 and the Jetson Nano.

The performance results, including the optimizations, are shown in Table 4. Optimization techniques improve the runtime performance of the models, obtaining real-time runtime performance in two of the three embedded devices evaluated in this work. The disparity estimation remains the same, without significant changes from the nonoptimized version of the models. Overall, the models run 20% faster on the Jetson Xavier and Jetson TX2, and around 10% faster in the Jetson Nano compared with results presented in Table 2. Additionally, Table 5 shows a detailed runtime speed evaluation over anytime stage 1 on the Jetson TX2, which is representative of what is happening on the other devices and models. Anytime stage 1 consists of four steps. In the first step, a CNN extracts features from each input image generating a matrix of *F* features of size F×nH×nW, where nH and nW are the scaled width *W* and height *H* of the input images. The results show that the optimized version runs around 20% faster. Cost-volume is the second step that compares pixels’ features between the two images using the L1 distance metric (Algorithm 1). Results show that the initial cost volume computation, using a custom CUDA kernel, runs up to 20 times faster than its naive implementation in Pytorch. Cost volume postprocessing, or cost aggregation, is the third step that refines the cost volume through 3D convolutions to generate an output matrix of size D×nH×nW, where *D* is the maximum possible scaled disparity. Obtained results show that the runtime speed performance on 3D convolutions remains the same—the latter due to the hardware and software limitations described in the previous section. Lastly, the regression is the final step, where a softargmin function obtains an output scaled disparity map of size nH×nW that later is upsampled through bilinear interpolation to its original size H×W. The regression runtime barely improves in the optimized version.

It is known that 3D convolutions are useful for dealing with volumetric data, such as the one from the cost-volume, thus they are commonly used on stereo disparity estimation models. However, 3D convolutions are much slower than 2D convolutions and are not currently suitable for fast implementations on models that run on embedded GPU devices. In the next section, we propose an alternative for postprocessing the cost-volume using 2D convolutions instead of 3D convolutions, improving the runtime significantly.

## 6. U-Net Like Model for Cost-Volume Postprocessing

From the evaluations in previous sections, we notice that:

- 3D convolutions are costly operations to run on current embedded GPU devices, at least when compared to 2D convolutions. Hence, it is necessary to look for alternatives to 3D convolutions, with a similar performance and a faster runtime speed.
- Reducing the runtime of cost-volume postprocessing can allow current models to compute disparity maps at a higher resolution.
- Many applications require continuous depth estimation, so multisteps models need to run over a fixed number of layers. Hence, it is preferable to set each model to its maximum capacity for the given embedded device. The latter improves the performance of the network since its loss functions do not need to trade off between the disparity pixel error over the different steps.

**Figure 2:**
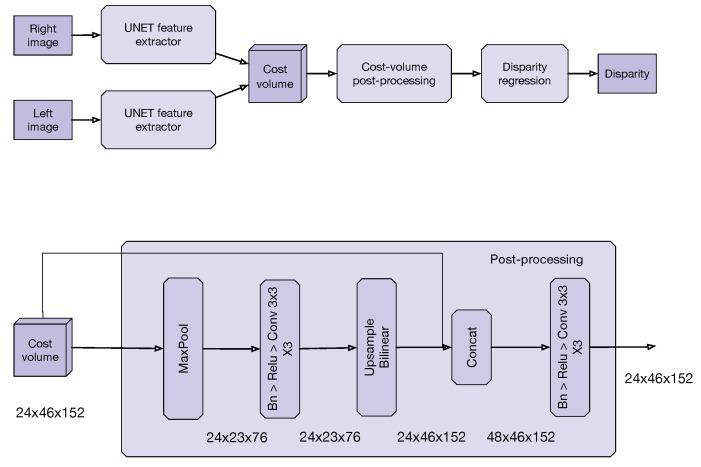
Final model. (**top**) First proposal, a single step stereo depth estimation model. (**bottom**) Second proposal, a cost volume postprocessing method using 2D convolutions instead of a sequence of 3D convolution layers.

According to the previous observations, we propose a sequential and a more simple model to estimate disparity maps from stereo images. Our model, based on [5], is depicted in Figure 2.

### 6.1. Model Description

Our model consists of five sequential steps:

- **Feature extraction**: The first step is to extract features from both input images using a U-Net [6] architecture. In essence, U-Net is an encoder-decoder network with skip connections between the downsampled and upsampled features that have the same dimension. In our case, we downsample the input image three times, reducing 16 times the original size (lines 1–12 in Table 6) and upsample once (lines 13–16 in Table 6). The upsampling process is key, and the number of upsampling steps will depend on the resources of the target device. For example, if we upsample once, the number of possible disparities to search became 192/8 (scaled disparity), where 192 is the maximum disparity. If we upsample twice, then the number of possible disparities became 192/4, generating a bigger cost-volume matrix (line 17 in Table 6). In our experiments, we choose one upsampling step to allow the Jetson Nano to run in real-time.
- **Cost-volume**: This is the second step and consists of relating features from both images, using an L1 distance metric. We use Algorithm 1.
- **Cost-volume postprocessing**: A U-Net model, like the one used for feature extraction, is used to postprocess the cost-volume instead of the traditional sequence of 3D convolutions. The intuition behind our proposal is that having an encoder-decoder model, that uses 2D convolutions for postprocessing the cost-volume matrix, helps to reduce the lack of volumetric insight that 3D convolutions have (lines 18–26 in Table 6). In our experiments, we downsample and upsample just once, since the resulting cost-volume matrix was small.
- **Disparity regression**: A softargmin [14] function determines the disparity as a weighted average of the output of the previous step (line 27 in Table 6).
- The last step is to upsample the disparity estimation to the original size, using bilinear interpolation (line 28 in Table 6).

### 6.2. Evaluation

We follow the same training and evaluation methodology from previous sections. We use the same smooth L1 loss function from [5], and features extraction occurs at 1/8 of the original input image size. We use Adam [21] as the optimizer, with learning rate 1e-3 and beta values of 0.9 and 0.999. Pytorch Lighting was used for the implementation of the model [22].

**Figure 3:**
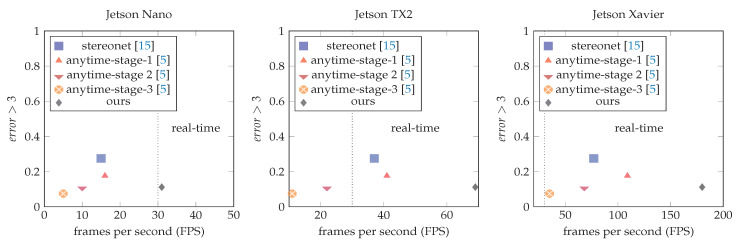
Comparative results of our method against state-of-the-art models. All models are optimized as in Section 5.

Table 7 shows the results of our experimental evaluation. From the table, we can see that baseline 3D—a sequence of 3D convolutions for the cost-volume postprocessing step—is the best solution in terms of pixel disparity error but inapplicable to embedded devices if real-time performance is required. Estimating the disparity at 1/8 of the original input size is too computationally costly using 3D convolutions (see Table 8). From Table 7, we can also visualize that a naive replacement of 3D convolutions for 2D convolutions increases the disparity pixel error almost two times. On the contrary, our proposed model achieves 11.2% of disparity pixel error and can even run on real-time speed on the Jetson Nano embedded GPU device. Our proposed model has a few more convolutions than the baseline 2D model, due to the U-Net architecture at the cost-volume postprocessing step, hence the slight difference in runtime. Comparative results with state-of-the-art methods are shown in Figure 3.

It is essential to notice that the results from Table 7 are much better in terms of runtime speed and pixel disparity error for the Jetson TX2 and Jetson Nano under real-time constraints. On the Jetson Xavier, results are 2% worst that anytime-stereo stage 3, but it runs almost six times faster. Further improvements could be achieved computing the disparity map at 1/4 of the original resolution.

## 7. Discussion

**Figure 4:**
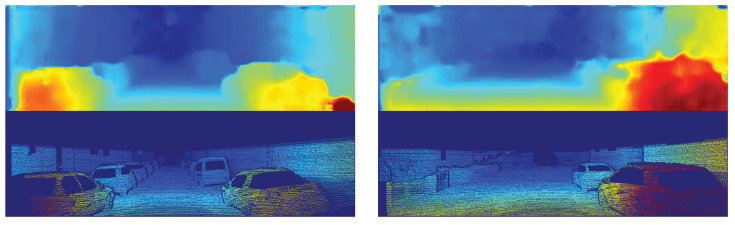
Examples of stereo disparity estimation using the proposed model (ground truths are depicted at the bottom of each image). (**left**) Disparity estimation over image pair 172 of the Kitti 2012 dataset [17]. (**right**) Disparity estimation over image pair 166 of the Kitti 2012 dataset [17] (in this case some artifacts appear in the estimated disparity map).

Although our proposal achieves a similar disparity pixel error performance to a sequence of 3D convolutions (see Figure 4 (left)), we notice that the resulting visual disparity tends to have artifacts in some images (see Figure 4 (right)). Future work might consider the use of an additional loss function for the cost-volume, to simplify the work of the postprocessing step (e.g., [4]).

In this work, we computed disparity initially at 1/8 of the original input resolution, upsampling later the disparity map to the original input size. We did this to show the potential of our idea on the Jetson Nano and the Jetson TX2 platforms. However, further improvements in pixel accuracy could be achieved with the Jetson Xavier, computing the disparity at a much higher resolution.

Finally, current models can potentially run faster on embedded GPU devices by using computation-efficient layers CNN architectures, like ghostnet [23] or shufflenet [24]; these architectures could be used in the models to reduce even more the inference runtime. Additionally, it will be interesting to try INT8 weight layers as future work. Hardware and operating system optimizations can be additionally tuned to improve the CNNs performance.

## 8. Conclusions

In this work, we evaluated two different state-of-the-art disparity network models on three different embedded GPU devices. We show that current models can run faster than stated in the literature, through the use of custom CUDA kernels and inference optimizations. Moreover, we show that further speed improvements can be achieved when 2D convolutions are used instead of 3D convolutions for cost-volume postprocessing. We perform inference at real-time speed, at most 33 ms per image pair, even in the Jetson Nano platform with 11% of disparity pixel error.

## Figures and Tables

**Table 1:** List of specifications for the embedded devices used in this work.

	Jetson TX2 (2017)	Jetson Xavier (2018)	Jetson Nano (2019)
**Architecture**	Pascal	Volta	Maxwell
**SM**	2	8	1
**CUDA cores per SM**	128	64	128
**CPU**	6 (4 + 2) core ARM	8 core ARM	4 core ARM
**Memory**	8 GB 128 bit LPDDR4	16 GB 256-bit LPDDR4x	4 GB 64-bit LPDDR4
**Bandwidth**	58.4 GB/s	137 GB/s	25.6 GB/s
**Storage**	32 GB eMMC	32 GB eMMC	16 GB eMMC 5.1 Flash
**Power**	7.5 W/15 W	10 W/15 W/30 W	5 W/10 W

**Table 2:** Runtime and error performance for anytime-stereo and stereonet models. Anytime stage 1 corresponds to anytime-stereo disparity estimation at 1/16 of the original resolution, anytime stage 2 at 1/8, and anytime stage 3 at 1/4. Stereonet was used without refinement steps at 1/16 of the original resolution. Real-time performance is marked in bold where lower is better. A Titan XP (Pascal) GPU is used as a reference of high-end desktop GPU performance. The disparity error (Err > 3) of each model (row) is the same in all devices.

Model	Err > 3	Titan XP	Jetson TX2	Jetson Xavier	Jetson Nano
**Anytime stage 1**	0.177	**4 ms**	39 ms	**13 ms**	77 ms
**Anytime stage 2**	0.115	**6 ms**	60 ms	**19 ms**	107 ms
**Anytime stage 3**	0.075	**10 ms**	105 ms	**33 ms**	200 ms
**Stereonet x16**	0.275	**4 ms**	45 ms	**17 ms**	96 ms

**Table 3:** On the top subtable, a list of available layers in the evaluated models and their support in TensorRT 6.X SDK. On the bottom subtable, a list of supported precision modes for the different embedded graphic processing unit (GPU) devices used in this work.

Available Layers	FP32	FP16	INT8	INT32	DLA FP16	DLA INT8	
**2D Conv**	Yes	Yes	Yes	No	Yes	Yes	
**3D Conv**	Yes	Yes	No	No	No	No	
**Batchnorm 2D**	Yes	Yes	Yes	No	Yes	Yes	
**Batchnorm 3D**	Yes	Yes	Yes	No	Yes	Yes	
**ReLU**	Yes	Yes	Yes	No	Yes	Yes	
**GPU Devices**	**CUDA**	**FP32**	**FP16**	**INT8**	**FP16 Tensor Cores**	**INT8 Tensor Cores**	**DLA**
**AGX Xavier**	7.2	Yes	Yes	Yes	Yes	Yes	Yes
**TX2**	6.2	Yes	Yes	No	No	No	No
**Nano**	5.3	Yes	Yes	No	No	No	No

**Table 4:** Runtime and error performance for stereonet and anytime-stereo models after optimization techniques are applied. Anytime stage 1 corresponds to anytime-stereo disparity estimation at 1/16 of the original resolution, anytime stage 2 at 1/8, and anytime stage 3 at 1/4. Stereonet was used without refinement steps at 1/16 of the original resolution. In black real-time performance, lower is better. The disparity error (Err > 3) for each model is the same in all devices.

Model	Err > 3	Jetson TX2	Jetson Xavier	Jetson Nano
**Anytime stage 1**	0.177	**24 ms**	**9 ms**	62 ms
**Anytime stage 2**	0.115	45 ms	**15 ms**	97 ms
**Anytime stage 3**	0.075	95 ms	**29 ms**	210 ms
**Stereonet x16**	0.275	**27 ms**	**13 ms**	65 ms

**Table 5:** Runtime performance of anytime stage 1 on Jetson TX2 with and without model optimizations.

Jetson TX2	Feature Extraction Mostly 2D Convs)	Cost-Volume (Matrix Operations)	Cost-Volume Post Processing (Mostly 3D Convs)	Regression (SoftArgMin)
**Anytime Stage 1** **without optimizations**	0.011921 s	0.011517 s	0.013466 s	0.002360 s
**Anytime Stage 1** **with optimizations**	0.009067 s	0.000346 s	0.013466 s	0.001414 s

**Table 6:** Proposed model configuration. Bn -> ReLU -> Conv 2D corresponds to BatchNorm 2D operation followed by a ReLU operations and finalizing with a convolution 2D with kernel size 3.

#	Layer (type)	Output Shape	# Params
**1**	Conv 2D 3 × 3	1 × 368 × 1218	28
**2**	BatchNorm 2D	1 × 368 × 1218	2
**3**	ReLU	1 × 368 × 1218	0
**4**	MaxPool 2D	1 × 92 × 304	0
**5**	Bn -> ReLU -> Conv 2D	2 × 92 × 304	20
**6**	Bn -> ReLU -> Conv 2D	2 × 92 × 304	40
**7**	MaxPool 2D	2 × 46 × 152	0
**8**	Bn -> ReLU -> Conv 2D	4 × 46 × 152	76
**9**	Bn -> ReLU -> Conv 2D	4 × 46 × 152	152
**10**	MaxPool 2D	4 × 23 × 76	0
**11**	Bn -> ReLU -> Conv 2D	8 × 23 × 76	296
**12**	Bn -> ReLU -> Conv 2D	8 × 23 × 76	592
**13**	Bilinear upsample	8 × 46 × 152	0
**14**	Concat 13 and 9	12 × 46 × 152	0
**15**	Bn -> ReLU -> Conv 2D	8 × 46 × 152	888
**16**	Bn -> ReLU -> Conv 2D	8 × 46 × 152	592
**17**	Cost-volume	24 × 46 × 152	0
**18**	MaxPool 2D	24 × 23 × 76	0
**19**	Bn -> ReLU -> Conv 2D	24 × 23 × 76	5232
**20**	Bn -> ReLU -> Conv 2D	24 × 23 × 76	5232
**21**	Bn -> ReLU -> Conv 2D	24 × 23 × 76	5232
**22**	Bilinear upsample	24 × 46 × 152	0
**23**	Concat 22 and 17	48 × 46 × 152	0
**24**	Bn -> ReLU -> Conv 2D	24 × 46 × 152	10,464
**25**	Bn -> ReLU -> Conv 2D	24 × 46 × 152	5232
**26**	Bn -> ReLU -> Conv 2D	24 × 46 × 152	5232
**27**	Soft argmin	1 × 46 × 152	0
**28**	Bilinear upsample	1 × 368 × 1218	0
		**Total**	39,319

**Table 7:** Final results of our proposed model. All models were optimized using TensorRT and using the custom CUDA kernel from Algorithm 1. Baseline 3D is the model from Figure 2 (top) but using a sequence of 3D convolutions for the cost-volume postprocessing. Baseline 2D is similar to baseline 3D but using 2D convolutions instead of 3D convolutions (naive approach). In black real-time performance; lower is better. The disparity error (Err > 3) for each model is the same in all devices. All models are optimized as in Section 5.

	Err > 3	Jetson TX 2	Jetson Xavier	Jetson Nano
**Baseline 3D**	0.090	162 ms	46 ms	439 ms
**Baseline 2D**	0.210	**14 ms**	**5 ms**	**32 ms**
**Proposed model**	0.112	**14 ms**	**5 ms**	**32 ms**

**Table 8:** Runtime performance of baseline 3D, baseline 2D, and the proposed model in the Jetson Nano. All models share the same architecture for the feature extraction, cost-volume, and regression steps. The cost-volume postprocessing architecture is different in all the models. All models are optimized as in Section 5.

Jetson Nano	Feature Extraction (mostly 2D convs)	Cost-Volume (Matrix Operations)	Cost-Volume Post Processing (Mostly 3D Convs)	Regression (SoftArgMin)
**Baseline 3D**	0.025766 s	0.000501 s	0.410672 s	0.002663 s
**Baseline 2D**	0.025766 s	0.000501 s	0.002911 s	0.002663 s
**Proposed model**	0.025766 s	0.000501 s	0.003136 s	0.002663 s
